# PP16 International Approaches To Integrating Sex And Gender In Health Technology Assessment

**DOI:** 10.1017/S0266462325102365

**Published:** 2025-12-29

**Authors:** Analía Abt Sacks, Anabel Granja Dominguez, Gustavo Mora, Yolanda Triñanes Pego, Eva Reviriego Rodrigo, Maria Jose Vicente-Edo

## Abstract

**Introduction:**

Sex differences and gender inequalities in health are increasingly recognized in health technology assessment (HTA), but their analysis remains inconsistent. Despite advancements in addressing ethical, organizational, and social issues in HTA, neglecting sex/gender (S/G) considerations can distort its outcomes. This initiative aimed to enhance equity by exploring international experiences and identifying frameworks for S/G-sensitive HTA processes.

**Methods:**

The first phase involved an exploratory analysis to assess the inclusion of S/G in methodological and report prioritization processes, using both Medical Subject Heading terms and free keywords. The search was then expanded to include international experiences, frameworks, and tools incorporating a S/G approach from organizations such as the World Health Organization, International Network of Agencies for Health Technology Assessment, European Network for Health Technology Assessment, Cochrane Collaboration, and other frameworks (GRADE, VALIDATE). HTA agencies and ministries of health from Latin America and high-income countries were reviewed. Data were extracted, focusing on the practical application of S/G in HTA.

**Results:**

No specific tools were identified, and S/G perspectives were generally absent, with “gender” mainly referring to the male/female distinction. The Cochrane Collaboration has a S/G Methods Group and a S/G in Systematic Reviews Planning Tool. Both the Cochrane Collaboration and GRADE incorporate S/G in equity frameworks (PROGRESS-Plus). Ecuador and Argentina (Instituto de Efectividad Clínica y Sanitaria) integrate gender in the Sustainable Development Goals. The National Institute for Health and Care Excellence includes S/G in qualitative study appraisals, and Canada’s Drug Agency (CDA) advocates for gender equity in health care. The Institute for Quality and Efficiency in Health Care (IQWiG) in Germany emphasizes the need to account for gender differences in health assessments as part of general HTA methods, and the French National Authority for Health (HAS) proposes integrating gender into public health policies and health technology guidance.

**Conclusions:**

S/G perspectives are internationally recognized as a key axis of inequity. Several entities incorporate these perspectives into documents and frameworks (the Cochrane Collaboration, CDA’s real-world evidence guidelines, IQWiG’s general methods). HAS plans methodological frameworks, and both the Institute for Clinical Effectiveness and Health Policy and IQWiG promote non-sexist/neutral language guidelines. The next exploratory phase would be to see whether and how they are being used in HTA processes.

